# Paralytic Ileus as the Initial Manifestation of New-Onset Type 1 Diabetes Without Diabetic Ketoacidosis: A Case Report

**DOI:** 10.7759/cureus.102039

**Published:** 2026-01-21

**Authors:** Shiori Ouchida, Reiko Saito, Nao Ohama, Mami Kuwamura, Reiji Fukano

**Affiliations:** 1 Department of Pediatrics, School of Medicine, University of Occupational and Environmental Health, Japan, Kitakyushu, JPN

**Keywords:** gastrointestinal dysmotility, initial manifestation, paralytic ileus, pediatrics, type 1 diabetes

## Abstract

Type 1 diabetes (T1D) is an autoimmune disease characterized by pancreatic β-cell destruction and absolute insulin deficiency. In pediatric patients, T1D typically presents with classic symptoms such as polydipsia, polyuria, weight loss, or diabetic ketoacidosis (DKA). Although gastrointestinal symptoms may occur at disease onset, paralytic ileus as an initial manifestation of T1D, particularly in the absence of DKA, is extremely uncommon. A 15-year-old boy presented with severe abdominal pain and vomiting and was diagnosed with paralytic ileus based on abdominal radiographic findings. Mild hyperglycemia at initial presentation was initially interpreted as transient. Conservative management led to improvement in gastrointestinal symptoms; however, persistent glycosuria was noted. After discharge, marked polydipsia and weight loss developed. Subsequent evaluation revealed severe hyperglycemia and elevated hemoglobin A1c (HbA1c) levels. Endocrinological assessment demonstrated positive anti-glutamic acid decarboxylase and anti-insulinoma-associated antigen-2 (IA-2) antibodies with reduced endogenous insulin secretion. Autoimmune T1D without DKA was diagnosed, and intensive insulin therapy was initiated, resulting in stable glycemic control. Paralytic ileus preceding the diagnosis of new-onset autoimmune T1D without DKA is an exceptionally rare presentation in pediatric patients.

## Introduction

Type 1 diabetes (T1D) is a chronic autoimmune disease characterized by pancreatic β-cell destruction and absolute insulin deficiency [1]. In children and adolescents, it typically presents with polydipsia, polyuria, and weight loss, and delayed diagnosis is associated with an increased risk of diabetic ketoacidosis (DKA) [2-4]. While early recognition can prevent acute metabolic complications, atypical initial presentations may contribute to diagnostic delay in pediatric practice.

New-onset type 1 diabetes may occasionally present with atypical or organ-specific symptoms, including gastrointestinal manifestations, before the development of classic symptoms such as polyuria or polydipsia. Gastrointestinal complaints, including abdominal pain, nausea, vomiting, and altered bowel motility, are common in individuals with diabetes mellitus (DM) and may obscure an underlying endocrine disorder, particularly in pediatric patients in whom functional gastrointestinal disorders are prevalent [5]. Consequently, endocrine etiologies may be overlooked when gastrointestinal symptoms dominate the initial clinical presentation.

Paralytic ileus is rare in adolescents and is most often associated with postoperative conditions, infections, electrolyte abnormalities, medications, or systemic metabolic disturbances. Although ileus has been reported in association with DKA or long-standing diabetes complicated by autonomic neuropathy, paralytic ileus as the initial manifestation of newly diagnosed T1D, especially in the absence of DKA, has been scarcely documented [6]. When unexplained ileus is accompanied by hyperglycemia or glycosuria, diabetes mellitus should be included in the differential diagnosis, and even mild hyperglycemia warrants careful metabolic evaluation.

Here, we report a rare case in which paralytic ileus preceded the diagnosis of autoimmune T1D without DKA, highlighting an atypical clinical presentation that underscores the importance of considering metabolic disorders in pediatric patients with unexplained gastrointestinal dysmotility.

## Case presentation

In October 2024, a 15-year-old boy presented with severe abdominal pain and vomiting. Approximately two months before admission, he suffered intermittent abdominal pain and visited a gastroenterology clinic, where constipation was suspected. Laxatives and increased fluid intake were prescribed, resulting in temporary symptom relief; however, abdominal pain gradually worsened despite daily bowel movements.

**Figure 1:**
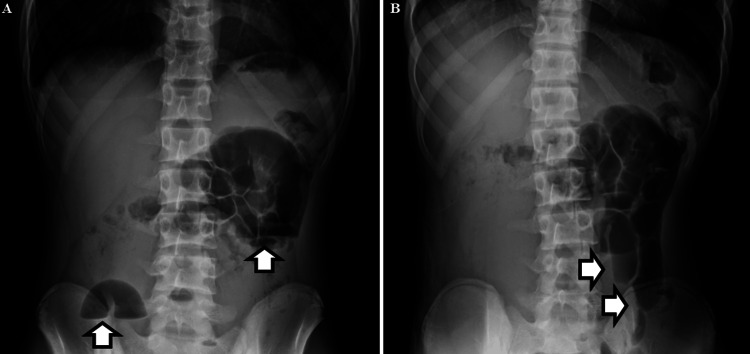
Plain abdominal radiographs that were obtained at initial presentation. (A) Upright view shows diffuse bowel gas distension with multiple air-fluid levels (arrows). (B) Right lateral decubitus view demonstrating marked intestinal gas accumulation without evidence of mechanical obstruction, consistent with paralytic ileus (arrows).

On the day of admission, the patient experienced severe abdominal pain accompanied by persistent vomiting and was unable to remain in a supine position. Abdominal radiography at a local clinic revealed diffuse bowel gas distension suggestive of ileus (Figure 1). White arrows indicate dilated bowel loops and air-fluid levels. His fasting blood glucose level was 196 mg/dL, which was interpreted as stress-associated hyperglycemia [7]. The patient was subsequently admitted to a local hospital.

Paralytic ileus was diagnosed at the local hospital, and conservative management with fasting and intravenous fluids improved the abdominal symptoms. Urinalysis on admission revealed glycosuria (3+) and ketonuria (3+). Although ketonuria resolved by discharge on day 7, glycosuria persisted. However, no further metabolic evaluation was performed, and the patient was discharged.

After discharge, marked polydipsia exceeding 2 L/day and progressive weight loss of approximately 3 kg over two months were evident. Three days later, follow-up evaluation revealed a random plasma glucose level of 421 mg/dL and a hemoglobin A1c (HbA1c) level of 9.9%. T1D was suspected, and the patient was referred to our hospital.

On admission, the patient's height and weight were 169.8 cm and 57.3 kg (BMI-standard deviation score {SDS}: -0.4), respectively. He was alert and hemodynamically stable, with no signs of Kussmaul respiration or severe dehydration. Physical examination revealed a flat and soft abdomen without tenderness or distension. Laboratory evaluation showed a plasma glucose level of 339 mg/dL and an HbA1c level of 9.9%. Venous blood gas analysis demonstrated a pH of 7.375 and a bicarbonate level of 24.3 mmol/L, indicating the absence of metabolic acidosis. Urinalysis showed a specific gravity of 1.048, glycosuria (4+), and ketonuria (+).

Endocrinological evaluation revealed positive anti-glutamic acid decarboxylase antibodies and anti-insulinoma-associated antigen-2 (IA-2) antibodies. Daily urinary C-peptide excretion was reduced, indicating impaired endogenous insulin secretion. Based on these findings, autoimmune T1D without DKA was diagnosed. Intensive insulin therapy using a basal-bolus regimen was initiated, resulting in the rapid stabilization of glycemic control. Abdominal symptoms did not recur after initiating insulin therapy, and the patient was discharged after completing diabetes education. During one year of outpatient follow-up after discharge, the patient remained asymptomatic with no recurrence of abdominal pain or vomiting. Glycemic control remained stable under intensive insulin therapy, and no further gastrointestinal complications have been observed to date. Laboratory findings at initial admission to the previous hospital are summarized in Table 1.

**Table 1:** Laboratory findings at initial admission to the previous hospital. HbA1c, hemoglobin A1c; HCO₃^-^, bicarbonate

Tests	Results	References
Fasting glucose	339 mg/dL	70-110
HbA1c	9.9%	4.6-6.2
Venous pH	7.375	7.35-7.45
HCO₃^-^	24.3 mmol/L	22-26
Sodium	136 mEq/L	135-145
Potassium	4.3 mEq/L	3.5-5.0
Chloride	100 mEq/L	98-108
Blood urea nitrogen	15.9 mg/dL	8-20
Creatinine	0.80 mg/dL	0.3-0.7
Anti-glutamic acid decarboxylase antibody	40 U/mL	<5.0
Anti-insulinoma-associated antigen-2 antibody	0.4 U/mL	<0.4

## Discussion

This case illustrates paralytic ileus as a rare initial manifestation of T1D in the absence of DKA. Although the precise pathophysiology remains unclear, the acute exacerbation of hyperglycemia may impair gastrointestinal motility even in early-stage diabetes.

The pathophysiological relationship between the acute exacerbation of hyperglycemia and gastrointestinal dysmotility is complex and multifactorial and may help explain the clinical course observed in this patient. In the present case, paralytic ileus improved transiently following conservative management at the previous hospital. Conservative management, including fasting and intravenous fluid replacement, may have partially ameliorated hyperglycemia, dehydration, and stress-related autonomic dysfunction, thereby contributing to temporary improvement in gastrointestinal motility. Importantly, this clinical improvement did not reflect the resolution of the underlying metabolic disturbance, as persistent glycosuria was noted despite symptomatic relief.

Several synergistic mechanisms likely contributed to the development of paralytic ileus in this patient. First, acute hyperglycemia can suppress gastrointestinal motility via neural pathways. High glucose levels may reduce cholinergic excitatory signaling from the vagus nerve [8] while enhancing the release of inhibitory neurotransmitters, such as nitric oxide, within the enteric nervous system [9]. Second, insulin deficiency and hyperglycemia disrupt gastrointestinal regulatory hormones. The abnormal secretion of peptides such as motilin, gastrin, and pancreatic polypeptide, essential for modulating intestinal transit, may have further compromised bowel movement in this diabetic state [5,10]. Third, hemodynamic factors can impair smooth muscle function. Severe hyperglycemia-induced osmotic diuresis leads to extracellular fluid volume depletion. This relative hypovolemia can compromise mesenteric microcirculation, reducing the contractile capacity of the intestinal smooth muscle. In our case, these factors likely acted synergistically to trigger overt paralytic ileus [11]. Notably, this occurred in the absence of metabolic acidosis or hypokalemia, which are the more common triggers in DKA-associated cases. This atypical presentation highlights paralytic ileus as a potential early manifestation of T1D. Other metabolic disorders that may cause gastrointestinal dysmotility, such as severe electrolyte imbalance, hypothyroidism, or inborn errors of metabolism, were considered less likely based on normal electrolyte levels, the acute clinical course, and the absence of supporting laboratory abnormalities.

Another notable aspect of this case is the absence of DKA at presentation. Subacute-onset T1D without metabolic acidosis may present with atypical or organ-specific symptoms, increasing the likelihood of diagnostic delay. While DKA often prompts the immediate consideration of DM, non-DKA presentations may be misattributed to other conditions. This case demonstrates that clinically significant gastrointestinal dysmotility may precede the diagnosis of T1D even in the absence of DKA.

## Conclusions

This case demonstrates that paralytic ileus can precede the diagnosis of new-onset autoimmune type 1 diabetes even in the absence of diabetic ketoacidosis. In this patient, gastrointestinal dysmotility was the dominant initial manifestation, preceding the development of overt hyperglycemia and classic diabetic symptoms.
